# Supplementary material for: Tissue of origin dictates GOT1 dependence and confers synthetic lethality to radiotherapy
Source: Cancer Metab. 2020 Jan 2;8:1. doi: 10.1186/s40170-019-0202-2 (PMC6941320; doi:10.1186/s40170-019-0202-2)
Supplement: Supplementary file 1 — Additional file 1. Supplementary figures. [file 40170_2019_202_MOESM1_ESM.pdf]

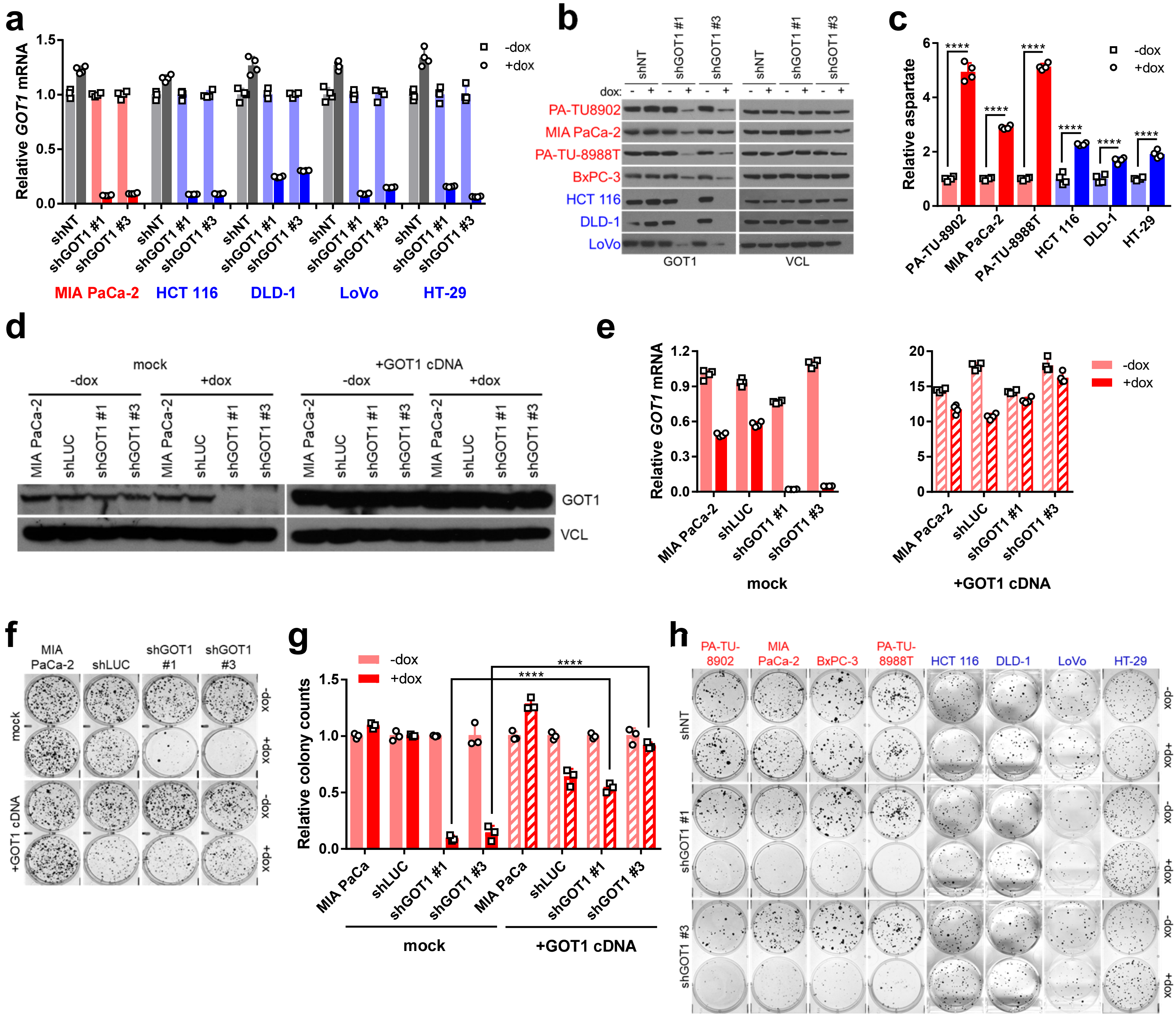

a
Relative GOT1 mRNA
-dox
+dox
shNT
shGOT1 #1
shGOT1 #3
MIA PaCa-2
HCT 116
DLD-1
LoVo
HT-29
b
dox:
PA-TU8902
MIA PaCa-2
PA-TU-8988T
BxPC-3
HCT 116
DLD-1
LoVo
GOT1
VCL
c
Relative aspartate
****
PA-TU-8902
MIA PaCa-2
PA-TU-8988T
HCT 116
DLD-1
HT-29
d
mock
+GOT1 cDNA
-dox
+dox
MIA PaCa-2
shLUC
shGOT1 #1
shGOT1 #3
GOT1
VCL
e
Relative GOT1 mRNA
mock
+GOT1 cDNA
f
MIA PaCa-2
shLUC
shGOT1 #1
shGOT1 #3
mock
+GOT1 cDNA
-dox
+dox
g
Relative colony counts
MIA PaCa
shLUC
shGOT1 #1
shGOT1 #3
mock
+GOT1 cDNA
h
PA-TU-8902
MIA PaCa-2
BxPC-3
PA-TU-8988T
HCT 116
DLD-1
LoVo
HT-29
shNT
shGOT1 #1
shGOT1 #3

E1

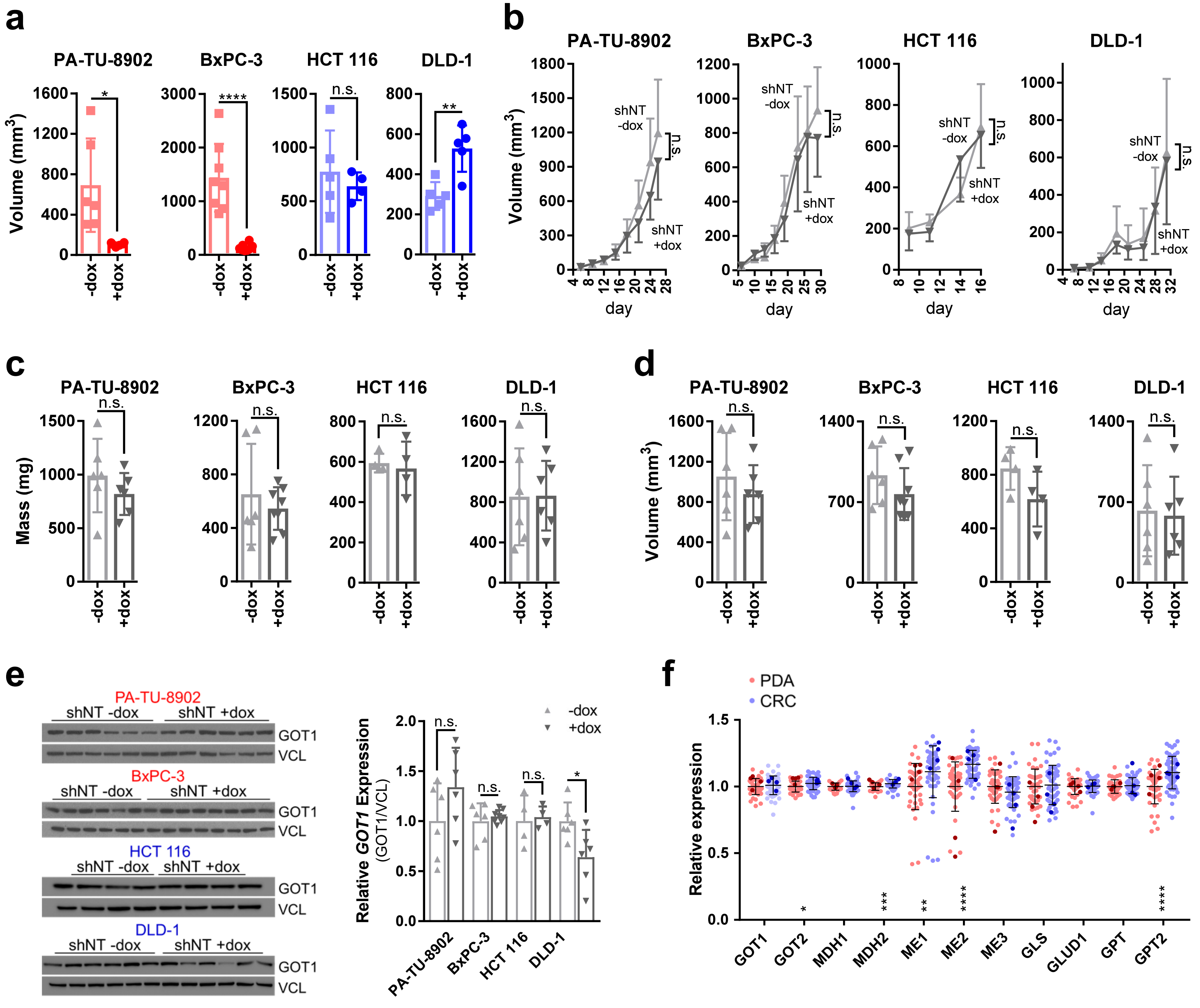
a
PA-TU-8902
BxPC-3
HCT 116
DLD-1
Volume (mm3)
-dox
+dox
*
****
n.s.
**
b
PA-TU-8902
BxPC-3
HCT 116
DLD-1
Volume (mm3)
shNT -dox
shNT +dox
n.s.
day
c
PA-TU-8902
BxPC-3
HCT 116
DLD-1
Mass (mg)
n.s.
-dox
+dox
d
PA-TU-8902
BxPC-3
HCT 116
DLD-1
Volume (mm3)
n.s.
-dox
+dox
e
PA-TU-8902
BxPC-3
HCT 116
DLD-1
shNT -dox
shNT +dox
GOT1
VCL
Relative GOT1 Expression
(GOT1/VCL)
-dox
+dox
n.s.
*
f
PDA
CRC
Relative expression
GOT1
GOT2
MDH1
MDH2
ME1
ME2
ME3
GLS
GLUD1
GPT
GPT2
*
***
**
****
****

E2

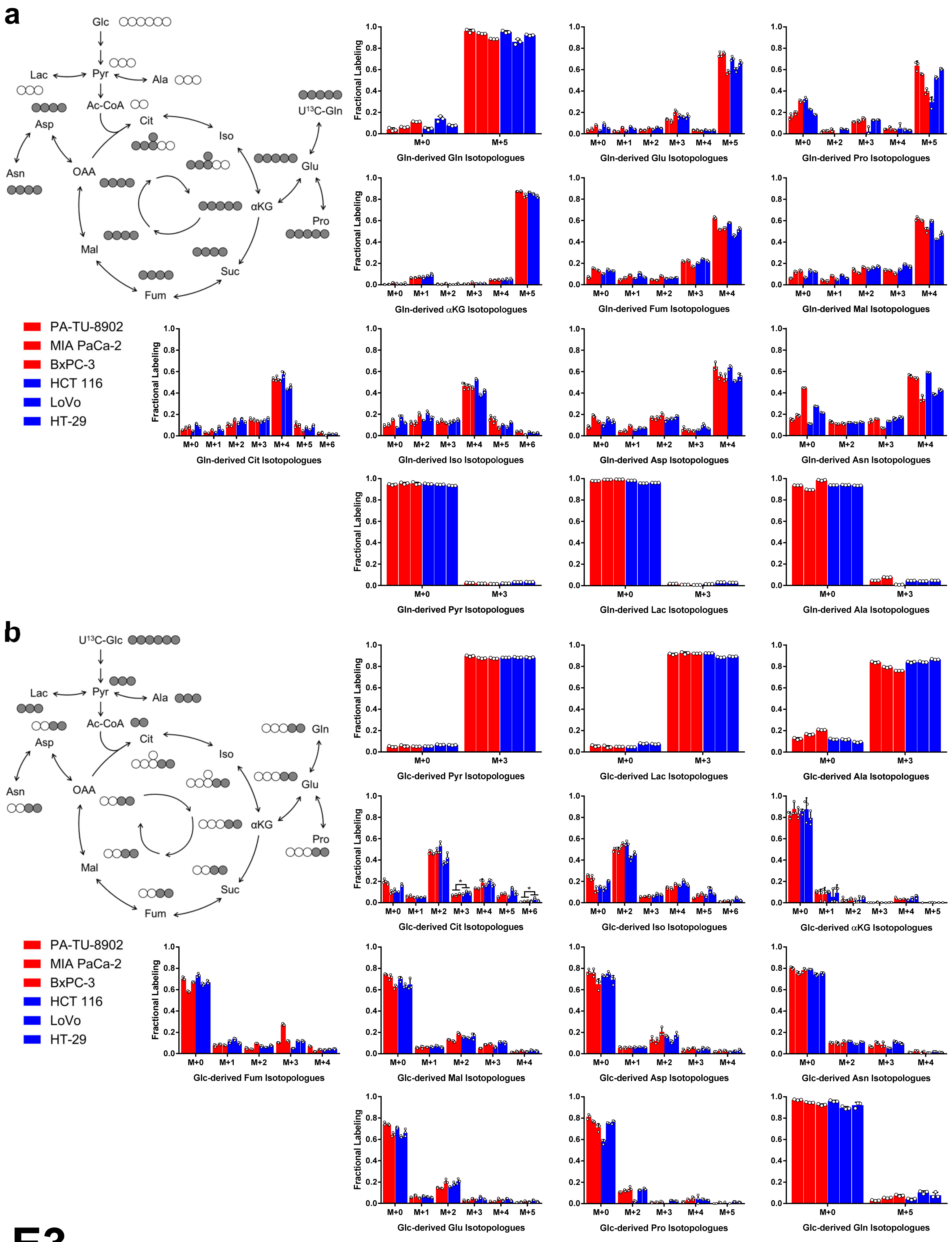
a
Glc
Lac
Pyr
Ala
Ac-CoA
Asp
Cit
U13C-Gln
Iso
Asn
OAA
Glu
αKG
Pro
Mal
Suc
Fum
PA-TU-8902
MIA PaCa-2
BxPC-3
HCT 116
LoVo
HT-29
Fractional Labeling
Gln-derived Gln Isotopologues
Gln-derived Glu Isotopologues
Gln-derived Pro Isotopologues
Gln-derived αKG Isotopologues
Gln-derived Fum Isotopologues
Gln-derived Mal Isotopologues
Gln-derived Cit Isotopologues
Gln-derived Iso Isotopologues
Gln-derived Asp Isotopologues
Gln-derived Asn Isotopologues
Gln-derived Pyr Isotopologues
Gln-derived Lac Isotopologues
Gln-derived Ala Isotopologues
b
U13C-Glc
Gln
Glc-derived Pyr Isotopologues
Glc-derived Lac Isotopologues
Glc-derived Ala Isotopologues
Glc-derived Cit Isotopologues
Glc-derived Iso Isotopologues
Glc-derived αKG Isotopologues
Glc-derived Fum Isotopologues
Glc-derived Mal Isotopologues
Glc-derived Asp Isotopologues
Glc-derived Asn Isotopologues
Glc-derived Glu Isotopologues
Glc-derived Pro Isotopologues
Glc-derived Gln Isotopologues

E3

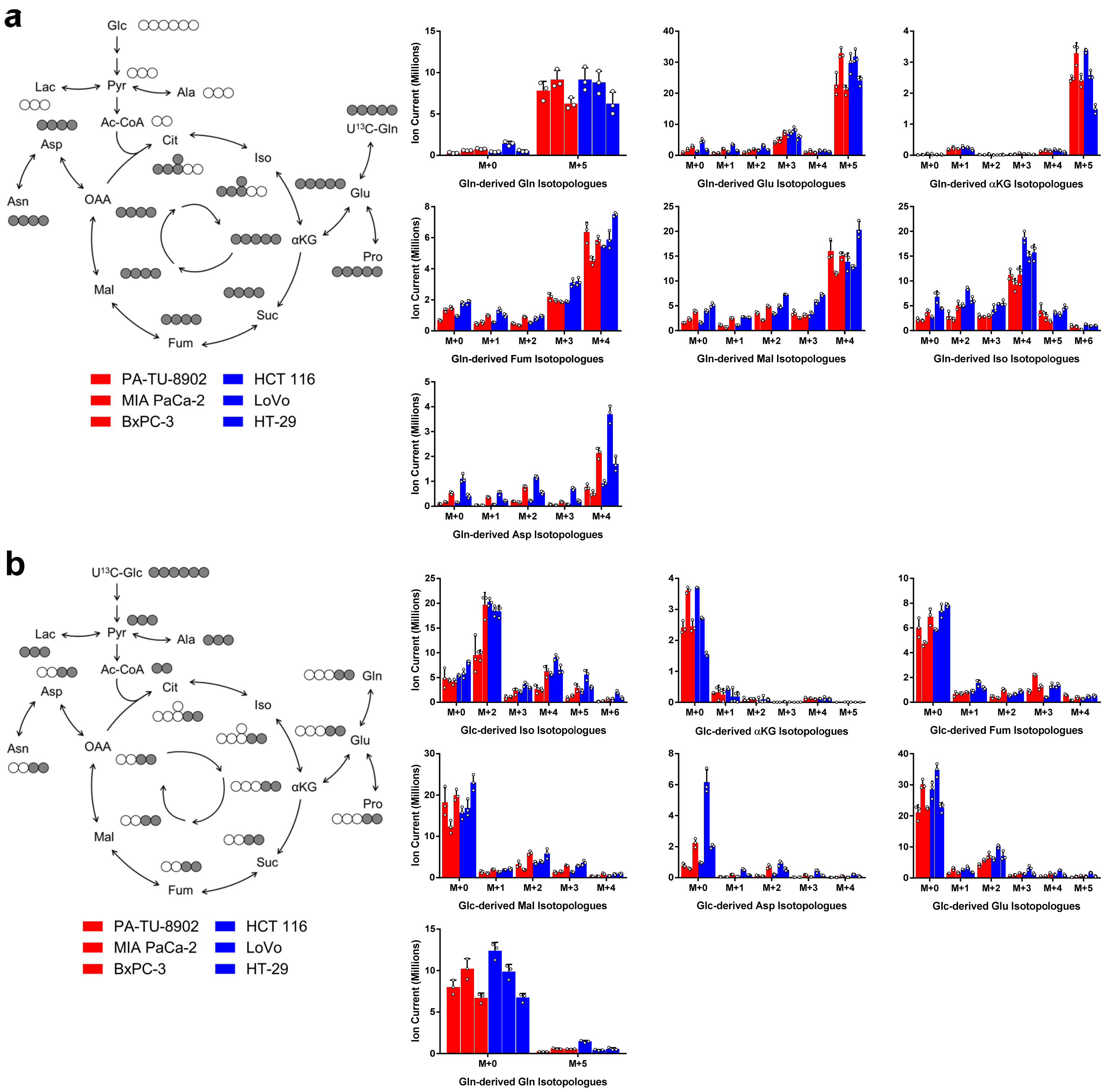
a
Glc
Lac
Pyr
Ala
Ac-CoA
Asp
Cit
Iso
U13C-Gln
Asn
OAA
Glu
αKG
Pro
Mal
Suc
Fum
PA-TU-8902
MIA PaCa-2
BxPC-3
HCT 116
LoVo
HT-29
Ion Current (Millions)
Gln-derived Gln Isotopologues
Gln-derived Glu Isotopologues
Gln-derived αKG Isotopologues
Gln-derived Fum Isotopologues
Gln-derived Mal Isotopologues
Gln-derived Iso Isotopologues
Gln-derived Asp Isotopologues
b
U13C-Glc
Gln
Glc-derived Iso Isotopologues
Glc-derived αKG Isotopologues
Glc-derived Fum Isotopologues
Glc-derived Mal Isotopologues
Glc-derived Asp Isotopologues
Glc-derived Glu Isotopologues
Gln-derived Gln Isotopologues

E4

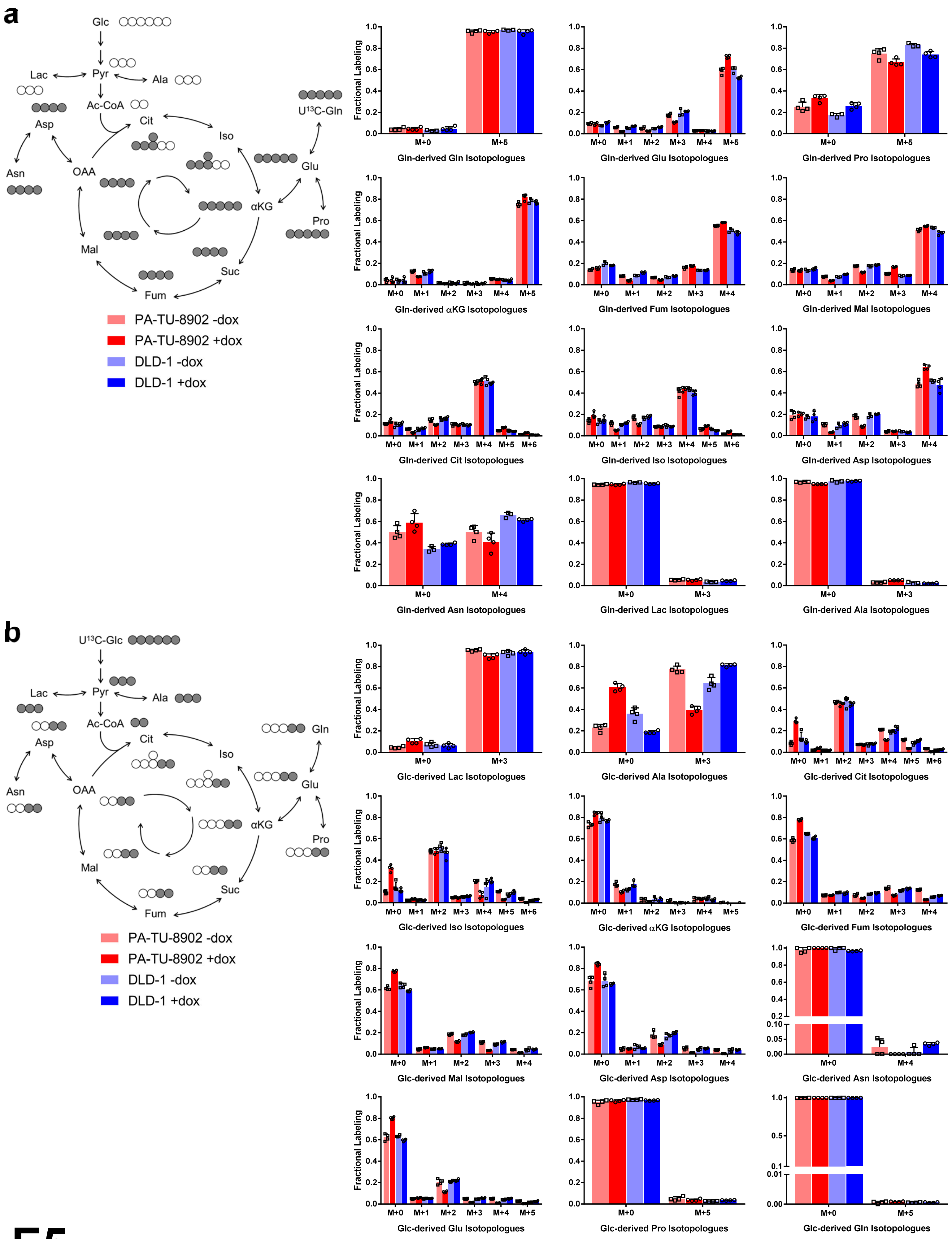
a
Glc
Lac
Pyr
Ala
Ac-CoA
Asp
Cit
U13C-Gln
Iso
Asn
OAA
Glu
αKG
Pro
Mal
Suc
Fum
PA-TU-8902 -dox
PA-TU-8902 +dox
DLD-1 -dox
DLD-1 +dox
Fractional Labeling
Gln-derived Gln Isotopologues
Gln-derived Glu Isotopologues
Gln-derived Pro Isotopologues
Gln-derived αKG Isotopologues
Gln-derived Fum Isotopologues
Gln-derived Mal Isotopologues
Gln-derived Cit Isotopologues
Gln-derived Iso Isotopologues
Gln-derived Asp Isotopologues
Gln-derived Asn Isotopologues
Gln-derived Lac Isotopologues
Gln-derived Ala Isotopologues
b
U13C-Glc
Gln
Glc-derived Lac Isotopologues
Glc-derived Ala Isotopologues
Glc-derived Cit Isotopologues
Glc-derived Iso Isotopologues
Glc-derived αKG Isotopologues
Glc-derived Fum Isotopologues
Glc-derived Mal Isotopologues
Glc-derived Asp Isotopologues
Glc-derived Asn Isotopologues
Glc-derived Glu Isotopologues
Glc-derived Pro Isotopologues
Glc-derived Gln Isotopologues

E5

a

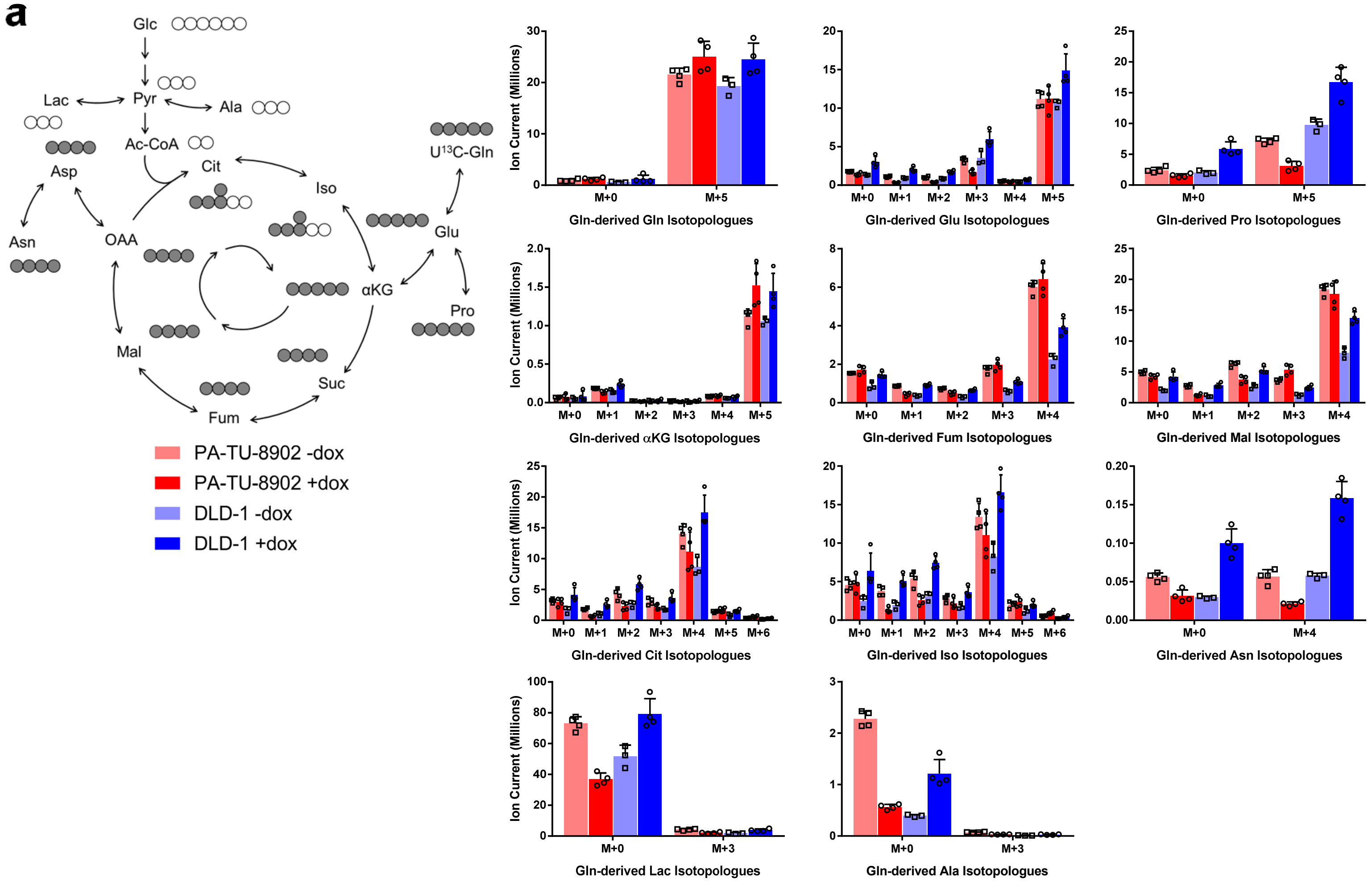

Glc
Pyr
Lac
Ala
Ac-CoA
Cit
Iso
Asp
Asn
OAA
αKG
Glu
U13C-Gln
Pro
Mal
Suc
Fum
PA-TU-8902 -dox
PA-TU-8902 +dox
DLD-1 -dox
DLD-1 +dox
Ion Current (Millions)
Gln-derived Gln Isotopologues
Gln-derived Glu Isotopologues
Gln-derived Pro Isotopologues
Gln-derived αKG Isotopologues
Gln-derived Fum Isotopologues
Gln-derived Mal Isotopologues
Gln-derived Cit Isotopologues
Gln-derived Iso Isotopologues
Gln-derived Asn Isotopologues
Gln-derived Lac Isotopologues
Gln-derived Ala Isotopologues

b

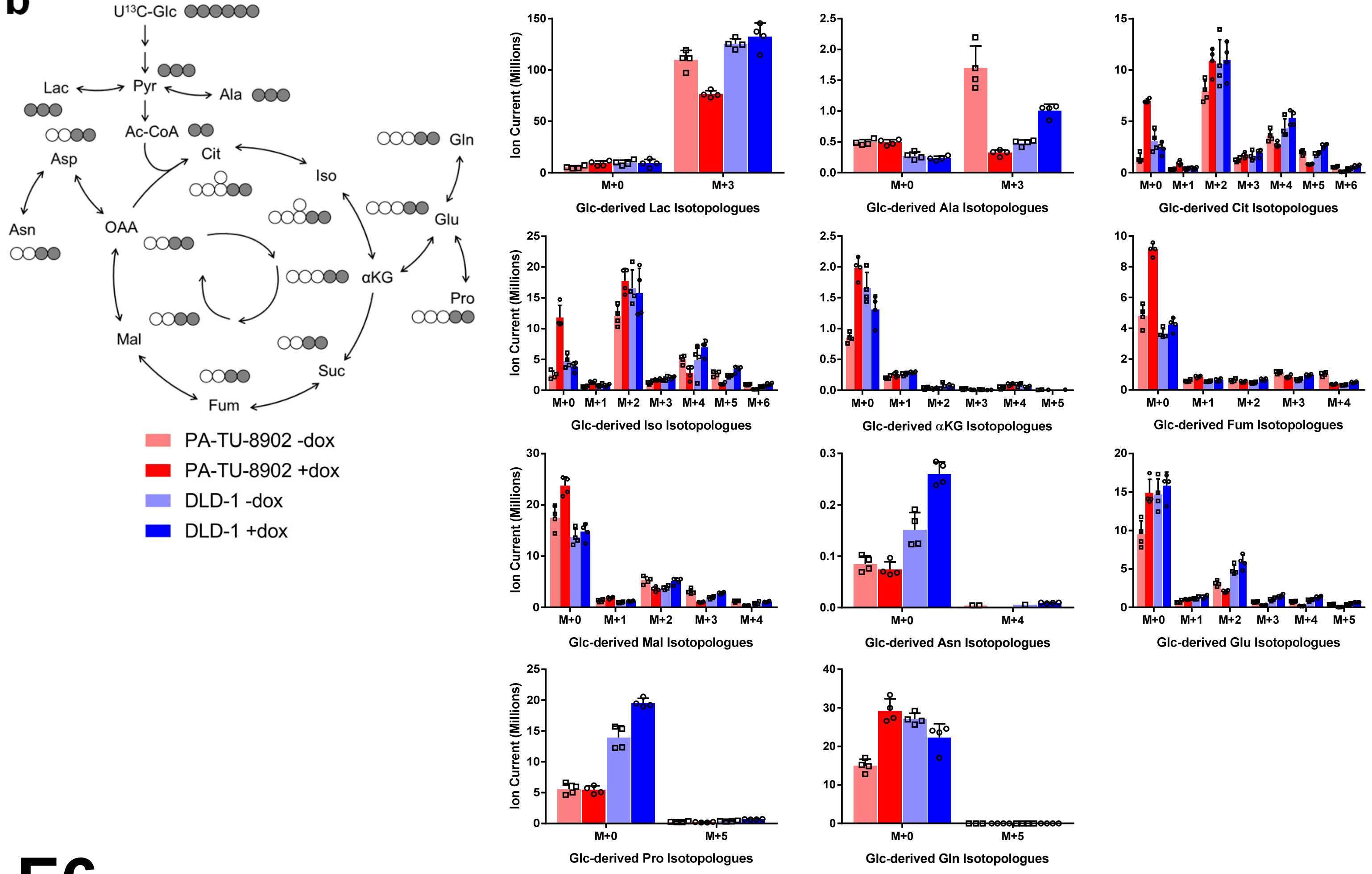

U13C-Glc
Pyr
Lac
Ala
Ac-CoA
Cit
Iso
Asp
Asn
OAA
Gln
Glu
αKG
Pro
Mal
Suc
Fum
PA-TU-8902 -dox
PA-TU-8902 +dox
DLD-1 -dox
DLD-1 +dox
Ion Current (Millions)
Glc-derived Lac Isotopologues
Glc-derived Ala Isotopologues
Glc-derived Cit Isotopologues
Glc-derived Iso Isotopologues
Glc-derived αKG Isotopologues
Glc-derived Fum Isotopologues
Glc-derived Mal Isotopologues
Glc-derived Asn Isotopologues
Glc-derived Glu Isotopologues
Glc-derived Pro Isotopologues
Glc-derived Gln Isotopologues

E6

a

Pathways Significantly Enriched upon GOT1 Knockdown in Vitro

Pathway Name	Hits	p	FDR
Pyrimidine metabolism	9/60	1.31E-06	1.05E-04
Purine metabolism	10/92	6.56E-06	2.62E-04
Cysteine and methionine metabolism	6/56	6.21E-04	0.0166
Sulfur metabolism	3/18	0.0046	0.0927
Nitrogen metabolism	4/39	0.0064	0.1018
Nicotinate and nicotinamide metabolism	4/44	0.0098	0.1304
Glycine, serine and threonine metabolism	4/48	0.0132	0.1402
Pantothenate and CoA biosynthesis	3/27	0.0147	0.1402
Arginine and proline metabolism	5/77	0.0158	0.1402
Glutathione metabolism	3/38	0.0365	0.2602
Cyanoamino acid metabolism	2/16	0.0376	0.2602
Glycerophospholipid metabolism	3/39	0.0390	0.2602

b

Pathways Significantly Enriched upon GOT1 Knockdown in Vivo

Pathway Name	Hits	p	FDR
Nicotinate and nicotinamide metabolism	5/44	2.26E-04	0.0181
Purine metabolism	6/92	0.0011	0.0430
Cysteine and methionine metabolism	4/56	0.0058	0.1553
Nitrogen metabolism	3/39	0.0141	0.2583
Aminoacyl-tRNA biosynthesis	4/75	0.0161	0.2583
Sulfur metabolism	2/18	0.0229	0.3060
Thiamine metabolism	2/24	0.0394	0.4346
Pyrimidine metabolism	3/60	0.0438	0.4346
Phenylalanine, tyrosine and tryptophan biosynthesis	2/27	0.0489	0.4346

c

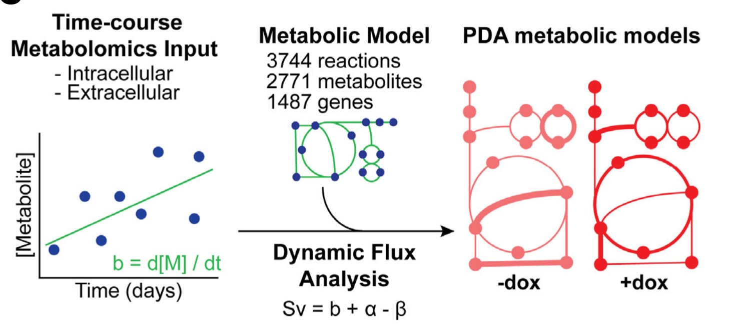

d

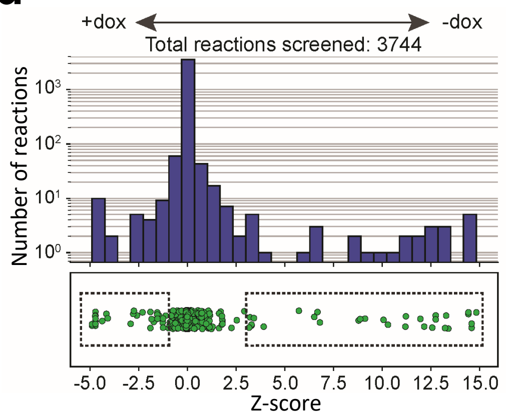

+dox

Urea Cycle

- argininosuccinate lyase
- argininosuccinate synthase
- ornithine carbamoyl-transferase

Pyrimidine Catabolism

- thymidine phosphorylase

-dox

Glycolysis

- phosphoglycerate mutase
- enolase
- fructose-bisphosphatase
- triose-phosphate isomerase
- fructose-bisphosphate aldolase
- phosphoglycerate kinase
- glyceraldehyde-3-phosphate dehydrogenase

Glutathione Metabolism

- glutathione transport into mitochondria
- gamma-glutamyltransferase
- gamma-glutamylcyclotransferase
- 5-oxoproline amidohydrolase

e

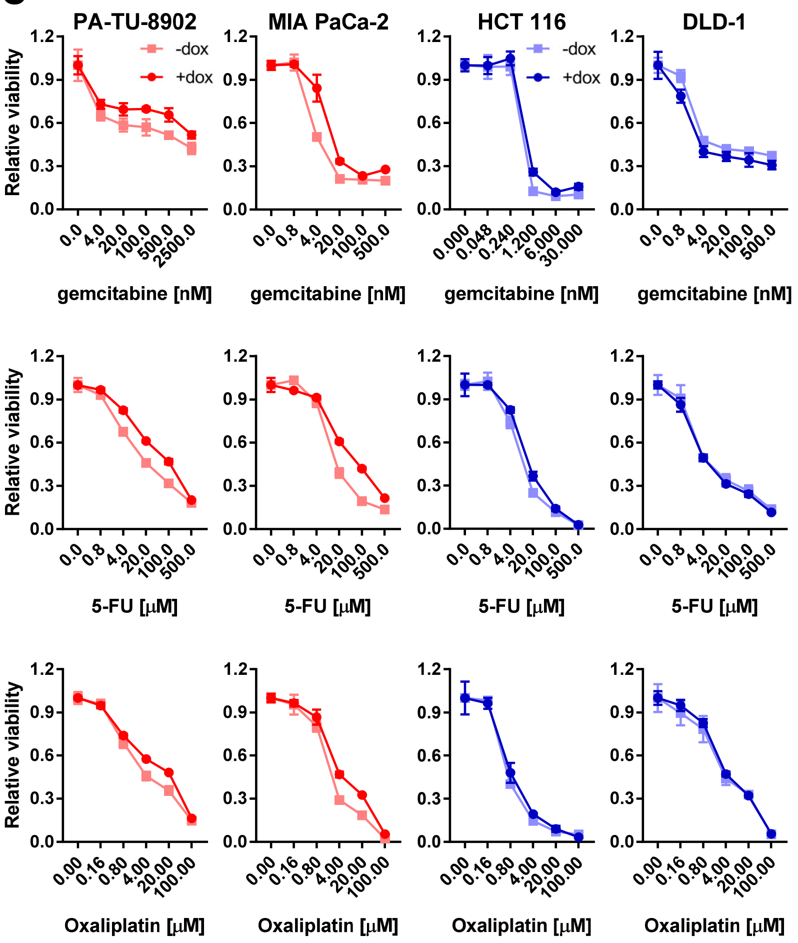

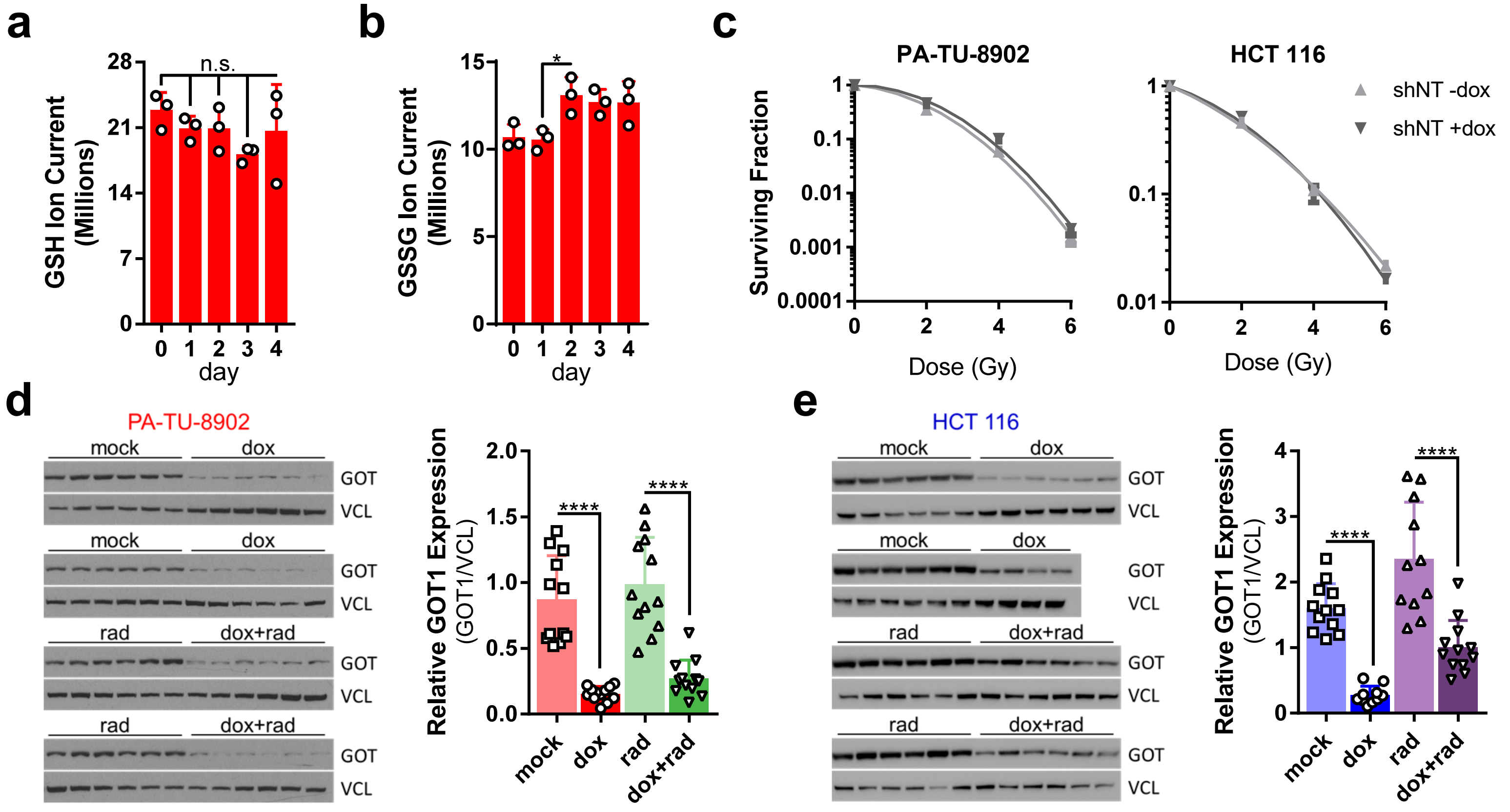
a
GSH Ion Current (Millions)
28
21
14
7
0
n.s.
0 1 2 3 4
day
b
GSSG Ion Current (Millions)
15
10
5
0
*
0 1 2 3 4
day
c
PA-TU-8902
HCT 116
Surviving Fraction
1
0.1
0.01
0.001
0.0001
0 2 4 6
Dose (Gy)
shNT -dox
shNT +dox
d
PA-TU-8902
mock
dox
rad
dox+rad
GOT
VCL
Relative GOT1 Expression (GOT1/VCL)
2.0
1.5
1.0
0.5
0.0
****
mock
dox
rad
dox+rad
e
HCT 116
4
3
2
1
0
